# Socioeconomic position and mental health care use before and after first redeemed antidepressant and time until subsequent contact to psychologist or psychiatrists: a nationwide Danish follow-up study

**DOI:** 10.1007/s00127-020-01908-7

**Published:** 2020-07-08

**Authors:** Aake Packness, Sonja Wehberg, Lene Halling Hastrup, Erik Simonsen, Jens Søndergaard, Frans Boch Waldorff

**Affiliations:** 1 Research Unit of General Practice, Department of Public Health, University of Southern Denmark, Odense, Denmark; 2 Psychiatric Research Unit, Psychiatry Region Zealand, Fælledvej 6, 4200 Slagelse, Denmark; 3 Department of Clinical Medicine, Faculty of Health and Medical Sciences, University of Copenhagen, Copenhagen, Denmark; 4 Section of General Practice and The Research Unit for General Practice, Department of Public Health, University of Copenhagen, Copenhagen, Denmark

**Keywords:** Socioeconomic factors, Mental health services, Access to health care, Antidepressants, Inequality

## Abstract

**Purpose:**

The purpose was to investigate inequalities in access to care among people with possible depression.

**Method:**

In this nationwide register-based cohort study of 30,593 persons, we observed the association between socioeconomic position (SEP, education/income) and mental health care use (MHCU) four months before the date of first redeemed antidepressant (Index Date/ID) and 12 months afterwards—and time to contact to psychologist/psychiatrist (PP). Logistic, Poisson, and Cox regression models were used, adjusted for sex, age, cohabitation, and psychiatric comorbidity.

**Results:**

Before ID, high SEP was associated with less GP contact (general practitioner), higher odds ratios for GP-Mental Health Counseling (GP-MHC), psychologist contact, and admissions to hospital. This disparity decreased the following 12 months for GP-MHC but increased for contact to psychologist; same pattern was seen for rate of visits. However, the low-income group had more contact to private psychiatrist.

For the 25,217 individuals with no MHCU before ID, higher educational level was associated with almost twice the rate of contact to PP the following 12 months; for the high-income group, the rate was 40% higher. 10% had contact to PP within 40 days after ID in the group with higher education; whereas, 10% of those with a short education would reach PP by day 120. High-income group had faster access as well.

**Conclusion:**

Being in high SEP was positively associated with MHCU, before and after ID, and more rapid PP contact, most explicit when measured by education. Co-payment for psychologist may divert care towards private psychiatrist for low-income groups.

**Electronic supplementary material:**

The online version of this article (10.1007/s00127-020-01908-7) contains supplementary material, which is available to authorized users.

## Introduction

Depression is a common disorder in high-income countries with the 12-month prevalence estimated at 5.5% and the lifetime prevalence at 14.6 [1]. Being in low socioeconomic position (SEP) is generally associated with higher morbidity [2], true for depressive disorders as well [3] with a dose–response relationship to income and education, and with higher incidence and stronger persistence of the disorder [4, 5]. Among others, childhood trauma, psychosocial impairment, older age, and socioeconomically disadvantaged status are found to be associated with proxies of need for highly specialized care of depression [6]. For mental health care, it is reported that only 22% of individuals in need receive minimally adequate treatment of depression in high-income countries [7], and being in low SEP is an additional risk for this [8]. It is of interest to know if these social inequalities in health care needs are reflected in the health care use.

Being highly prevalent, strongly associated with SEP, having a lifelong impact, and associated with considerable disability, and reduced life expectancy [9], make depression a relevant and good indicator to examining potential social inequality in mental health care.

WHO Europe defines equity in health care as equal access to available care for equal need, equal utilization for equal need, and equal quality of care for all [10]. Thus, in evaluating potential inequality in health care, it is not enough to observe usage: the need must be defined as well. In the literature, need is usually defined either as the patient’s perceived need or as clinical need—commonly defined by a questionnaire, often WHO’s International Diagnostic Interview (CIDI) [11], and health care use is reported by recall. We used the prescription of antidepressants as a professionally evaluated proxy for need and observed subsequent health care use in national registers.

Previously, we found high SEP positively associated with the use of specialized health care services following initiation of treatment with antidepressants; we also previously found that distance to services adds to inequality in use [12]. However, in evaluating the results, we became aware of shortcomings in the design. Not all patients initiate their antidepressant treatment with medications; some begin by consulting a psychologist first and this can be strongly associated with SEP, as can more rapid access to specialized care afterwards. These shortcomings were both possible to correct, extracting from the existing data. Ability to pay for care and ability to access care may both have an impact on mental health care use.

The purpose of this study was to investigate inequalities in access to care among people with possible depression by exploring differences in health care treatment between socioeconomic groups before and after initiating treatment with antidepressants and the timeliness of accessing specialized care afterwards.

## Method

### Study design

The study was conducted as a register-based follow-up study on general practitioner (GP) and mental health care use (MHCU) 4 months preceding date of first redeemed antidepressant, the index date (ID) and additional 12 months afterwards. GPs do not share information on diagnoses with public registers; to overcome this, the initiated use of antidepressant medication served as a proxy for need, as redeemed prescriptions are recorded in registers.

The Danish health care system is tax funded and free at delivery for both primary and secondary care except for dental care and treatments at psychologists, which are only partly subsidized for adults [13]. The GP has a gatekeeper function and specialized care is only free after referral. Treatment by a psychologist is subsidized for patients referred from a GP for some specific conditions: reaction to specific traumatic events, moderate depression and, specifically for citizens between 18 and 38 years old, also moderate anxiety disorders. According to the Danish treatment guidelines, patients with severe depressive disorders are to be referred to a psychiatrist. In 2014, the co-payment was equivalent to 52€ for the first consultation and 44€ for the following sessions [14]. The municipality can cover the co-payment if the patient has no means and the treatment is necessary to obtain or uphold a job.

1.9 million Danes (50% of the population aged 20–70 years) had a supplementary private health insurance plan by 2016, usually paid by the employer. Less than 3% of the insurance plans were privately paid. Expenses for psychiatric and psychologist treatment were 31.5€ million by 2016, which is an increase of 33% since 2013 [15]. The insurance only covers the co-payment; whereas, the public part of the expense for a psychologist (or a psychiatrist) are still public. Thus, privately insured persons are included in the national data.

The data extraction is described in detail in our previous study [12], and a general description of Danish registers relevant for public health and health care can be found elsewhere [16] why parts the description here is done short.

### Study population and study period

The study population comprised all individuals aged 20–64 years living in Denmark who were prescribed and redeemed antidepressants (Anatomical Therapeutic Chemical (ATC) classification system N06A) in 2013 between May and December, according to the data extracted from The Danish National Prescription Registry [17, 18]. Only patients with no previous prescription of antidepressants in 2012 were included. Bupropion (ATC N06AX12) was not included since it is only prescribed for smoking cessation in Denmark. Tricyclic antidepressants (ATCs N06AA) were not included either as they are not recommended as the first choice for treatment of depression or are frequently used as a secondary analgesic [19, 20]. All persons migrating in 2012 were excluded as they could not be accounted for during the full study period. Finally, all patients coded as terminally ill at first prescription, and thereby specially subsidized, were excluded, [21] since their mobility and ability to access care would be expected to be low and the prescription of antidepressants the last three months of life is known to be very high [22].

Data on health care use were extracted for 2013–2014. The individuals redeeming prescriptions the first 4 months of 2013 were not included in the analyses, since data on their 4 months previous use of health care were lacking. The resulting population was monitored for 16 months per individual—4 months before and twelve months after first redeemed prescription of antidepressant. Four months was chosen with the expectation that treatment with medication would be relevant at the 4-month mark if consulting a psychologist or GP for counseling did not work within 3–4 months.

The individuals were followed in the relevant public registers by the use of the 10-digit personal identification number from the Danish Civil Registration System (CRS) [16].

### Independent variables

Data on family income were drawn from the Registers of income. In this study, we used equivalent disposable family income [23]. Highest completed educational level was drawn from the Population’s Education Register [16]. The education is presented in years “ < 10 years” equals no post-secondary; “10–12 years” equals 1–3 years post-secondary and “ > 12 years” equals 3 + years post-secondary education. The term SEP would cover both education and income.

Data concerning age, sex, address, marital status, cohabitation status, and vital status were gathered from the CRS.

Information on comorbidity was drawn from The Danish National Patient Register and The Danish Psychiatric Central Research Register [16] (See Supplementary).

### Dependent variables

Data on the utilization of private psychiatrist, psychologist, and general practitioner (GP) were drawn from The Danish National Health Service Register for Primary Care and date of redeemed prescriptions of antidepressants from The Danish National Prescription Registry.

GP contact and mental health counseling by GPs (GP-MHC) were analyzed. GP-MHC covers talk therapy by a GP. It consists of at least two talks within the first 6 months and not more than seven talks within one year. The service triggers additional pay (See Supplementary to method).

Information on public inpatient and outpatient mental health care contacts was drawn from The Danish National Patient Register; ICD-10 coded F00 – F99.

One-day psychiatric hospital admissions were re-categorized into emergency contacts and termed as: Emergency and short admissions.

The collection and handling of the data have been approved by The Danish Data Protection Agency J. no. 2015-41-3984. Approval by an ethic committee is not required for register studies.

### Statistical analyses

Logistic regression analysis was used to estimate both the uni- and multivariable odds ratio (OR) for the association between SEP and each type of contact to a health service provider. To analyze how those who gained contact to a mental health service provider used the services, Poisson regression was used to estimate the uni- and multivariable incidence rate ratio (IRR) for the SEP. Both analyses were adjusted for binary sex, categorical age group, binary cohabitation status, and binary psychiatric comorbidity. Post hoc tests on associations with gender were performed.

Finally, we analyzed the time (days) to first contact of either psychologist or psychiatrist after first AD prescription, excluding patients who had reached the treatment level 4 and above before their first AD prescription. We present Kaplan–Meier graphs for failure for each category of SEP and use Cox regression models to estimate uni- and multivariable hazard ratios (HR). Censoring is noted at the time of death, emigration or day 365, adjusted for binary sex, categorical age group, binary cohabitation status, and binary psychiatric comorbidity.

OR, IRR, and HR (Hazard Ratio) were estimated at 95% confidence intervals (CI) and *p* values were reported.

## Results

53,515 individuals in the selected age group had a first prescription of AD in Denmark in 2013, and no AD prescription in 2012; of these, 20,935 had the prescription before May and were, thus, excluded. The resulting 32,580 individuals were reduced to 30,593 due to: age < 20 (N 1327), death before ID (N 1); migration in or out of the country in 2012 (N 405); not registered in the country at ID (N 66); Terminal patient (N 188).

In the year following the ID, some died (*N* = 332) and some emigrated (*N* = 121). The sample was followed in total 14,744,394 days.

57% of the study sample were female (Table 1), compared to 50% of the national population in the same age group. Compared to the national proportion of 10% with less than 10-year education, the sample had a three times higher proportion; the proportion temporally not in work (18%) was twice the national level. A higher proportion of females had a higher education and were cohabitating compared to men and the national population. Of the total of 30,593 individuals included, 5376 (18%) had mental health care treatment beyond GP contact before ID; the rest initiated their treatment with an antidepressant medication.

**Table 1:** Characteristics of the study sample before and after index date, national comparisons

	Treatment related to index date					
	All	Female	Male	DK^a^	Before^b^	After
	30593 (100.0)	17457 (57.1)	13136 (42.9)		5376 (17.6)	25217 (82.4)
Age group						
20–29	6722 (22.0)	3959 (22.7)	2763 (21.0)	20.9	1657 (24.7)	5065 (75.3)
30–39	7183 (23.5)	4266 (24.4)	2917 (22.2)	21.4	1406 (19.6)	5777 (80.4)
40–49	7674 (25.1)	4299 (24.6)	3375 (25.7)	25.0	1207 (15.7)	6467 84.3)
50–59	6588 (21.5)	3605 (20.7)	2983 (22.7)	22.3	882 (13.4)	5706 (86.6)
60–64	2426 (7.9)	1328 (7.6)	1098 (8.4)	10.5	224 (9.2)	2202 (90.8)
Education						
< 10 years	9992 (32.7)	5341 (30.6)	4651 (35.4)	10	1760 (17.6)	8232 (82.4)
10-12 years	12766 (41.7)	7125 (40.8)	5641 (42.9)	62	2140 (16.8)	10626 (83.2)
12 + years	6569 (21.5)	4357 (25.0)	2212 (16.8)	15	1275 (19.4)	5294 (80.6)
Not available	1266 (4.1)	634 (3.6)	632 (4.8)	4	201 (15.9)	1065 (84.1)
Income						
Lower 33%	10114 (33.1)	5529 (31.7)	4585 (34.9)		2095 (20.7)	8019 (79.3)
Middle quantile	10419 (34.1)	6101 (34.9)	4318 (32.9)		1765 (16.9)	8654 (83.1)
Upper 33%	10060 (32.9)	5827 (33.4)	4233 (32.2)		1516 (15.1)	8544 (84.9)
Employment status						
Employed/student	19765 (64.6)	11352 (65.0)	8413 (64.0)	79	3295 (16.7)	16470 (83.3)
Not employed	5554 (18.2)	3118 (17.9)	2436 (18.5)	9	1182 (21.3)	4372 (78.7)
Retired	3887 (12.7)	2189 (12.5)	1698 (12.9)	9	620 (16.0)	3267 (84.0)
Other	1387 (4.5)	798 (4.6)	589 (4.5)	3	279 (20.1)	1108 (79.9)
Cohabitating						
Single	12944 (42.3)	6846 (39.2)	6098 (46.4)	45	2646 (20.4)	10298 (79.6)
Cohabitating	17649 (57.7)	10611 (60.8)	7038 (53.6)	55	2730 (15.5)	14919 (84.5)
Number of chronic somatic conditions						
0-	26861 (87.8)	15564 (89.2)	11297 (86.0)		4879 (18.2)	21982 (81.8)
1-	3239 (10.6)	1653 (9.5)	1586 (12.1)		451 (13.9)	2788 (86.1)
2-	493 (1.6)	240 (1.4)	253 (1.9)		46 (9.3)	447 (90.7)
Comorbidity psychiatric						
No	22940 (75.0)	13211 (75.7)	9729 (74.1)		2594 (11.3)	20346 (88.7)
Yes	7653 (25.0)	4246 (24.3)	3407 (25.9)		2782 (36.1)	4871 (63.6)
Treatment before index date¤						
No	25217 (82.4)	14337 (82.1)	10880 (82.8)			
Yes	5376 (17.6)	3120 (17.9)	2256 (17.2)			
	30593 (100.0)					

^a^Statistics Denmark: www.statistikbanken.dk accessed august 2016. Data of age group 20–64 years as of January 2013

^b^Row percentage mental health care treatment before the date of first redeemed antidepressant

Before ID, high SEP (education and income) was associated with less contact to GP, higher aOR for contact to GP-MHC (aOR 1.8 and 1.5), to psychologist (aOR 2.6 and 2.2), and to admission to hospital care (Table 2). Except for GP, the aORs for contact to these services were still higher for individuals in high SEP after 16 months, decreasing for GP-MHC and increasing for psychologist contact.

**Table 2:** Education and income. Odds ratio for health care contact within 4 months before index date and total 16 months

	Education				
*N*	29327	OR adjusted (CI)		29327	OR adjusted (CI)
GP					
< 10 years	7722/9992 (77.3)	Ref		9818/9992 (98.3)	Ref
10–12 years	9710/12766 (76.1)	0.9 (0.9–1.0)		12595/12766 (98.7)	1.1 (0.9–1.4)
12+ years	4889/6569 (74.4)	0.8 (0.7–0.8)		6410/6569 (97.6)	0.5 (0.4–0.7)
GP-MHC					
< 10 years	943/9992 (9.4)	Ref		3259/9992 (32.6)	Ref
10–12 years	1543/12766 (12.1)	1.3 (1.2–1.4)		5017/12766 (39.3)	1.3 (1.2–1.4)
12+ years	1068/6569 (16.3)	1.8 (1.6–2.0)		2818/6569 (42.9)	1.5 (1.4–1.6)
Psychologist					
< 10 years	410/9992 (4.1)	Ref		1216/9992 (12.2)	Ref
10–12 years	897/12766 (7.0)	1.7 (1.5–2.0)		2699/12766 (21.1)	1.9 (1.7–2.0)
12+ years	669/6569 (10.2)	2.6 (2.3–3.0)		1861/6569 (28.3)	2.8 (2.5–3.0)
Private psychiatrist					
< 10 years	391/9992 (3.9)	Ref		1004/9992 (10.0)	Ref
10-12 years	386/12766 (3.0)	0.8 (0.7–1.0)		1088/12766 (8.5)	0.9 (0.8–1.0)
12+ years	242/6569 (3.7)	1.1 (0.9–1.3)		711/6569 (10.8)	1.2 (1.1–1.3)
Outpatient psychiatry					
< 10 years	583/9992 (5.8)	Ref		2039/9992 (20.4)	Ref
10–12 years	516/12766 (4.0)	1.3 (1.1–1.4)		2029/12766 (15.9)	1.0 (0.9–1.1)
12+ years	214/6569 (3.3)	1.3 (1.1–1.5)		872/6569 (13.3)	0.9 (0.9–1.0)
Emergency, short admission					
< 10 years	370/9992 (3.7)	Ref		637/9992 (6.4)	Ref
10–12 years	396/12766 (3.1)	1.2 (1.0–1.4)		704/12766 (5.5)	1.1 (1.0–1.3)
12+ years	184/6569 (2.8)	1.3 (1.0–1.5)		320/6569 (4.9)	1.2 (1.0–1.3)
Hospital admission					
< 10 years	282/9992 (2.8)	Ref		604/9992 (6.0)	Ref
10–12 years	250/12766 (2.0)	1.1 (0.9–1.4)		644/12766 (5.0)	1.2 (1.0–1.3)
12+ years	126/6569 (1.9)	1.3 (1.0–1.7)		311/6569 (4.7)	1.3 (1.1–1.5)

	Income				
*N*	30593	OR adjusted (CI)	30593		OR adjusted (CI)
GP					
Lower 33%	7681/10114 (75.9)	Ref	9909/10114 (98.0)		Ref
Middle quantile	8062/10419 (77.4)	1.0 (0.9–1.1)	10270/10419 (98.6)		1.2 (1.0–1.5)
Upper 33%	7535/10060 (74.9)	0.8 (0.8–0.9)	9869/10060 (98.1)		0.8 (0.6–1.0)
GP-MHC					
Lower 33%	1108/10114 (11.0)	Ref	3512/10114 (34.7)		Ref
Middle quantile	1214/10419 (11.7)	1.2 (1.0–1.3)	3886/10419 (37.3)		1.2 (1.1–1.2)
Upper 33%	1344/10060 (13.4)	1.5 (1.3–1.6)	4070/10060 (40.5)		1.4 (1.3–1.5)
Psychologist					
Lower 33%	507/10114 (5.0)	Ref	1435/10114 (14.2)		Ref
Middle quantile	700/10419 (6.7)	1.6 (1.4–1.8)	2014/10419 (19.3)		1.6 (1.5–1.8)
Upper 33%	804/10060 (8.0)	2.2 (1.9–2.5)	2435/10060 (24.2)		2.4 (2.2–2.6)
Private psychiatrist					
Lower 33%	533/10114 (5.3)	Ref	1310/10114 (13.0)		Ref
Middle quantile	307/10419 (2.9)	0.6 (0.5–0.7)	850/10419 (8.2)		0.6 (0.6–0.7)
Upper 33%	228/10060 (2.3)	0.5 (0.4–0.6)	755/10060 (7.5)		0.6 (0.5–0.7)
Outpatient psychiatry					
Lower 33%	692/10114 (6.8)	Ref	2262/10114 (22.4)		Ref
Middle quantile	455/10419 (4.4)	1.0 (0.8–1.1)	1705/10419 (16.4)		0.9 (0.9–1.0)
Upper 33%	235/10060 (2.3)	0.9 (0.8–1.1)	1175/10060 (11.7)		0.9 (0.8–1.0)
Emergency, short admission					
Lower 33%	416/10114 (4.1)	Ref	703/10114 (7.0)		Ref
Middle quantile	324/10419 (3.1)	1.1 (0.9–1.3)	565/10419 (5.4)		1.1 (1.0–1.2)
Upper 33%	257/10060 (2.6)	1.3 (1.1–1.6)	466/10060 (4.6)		1.3 (1.1–1.5)
Hospital admission					
Lower 33%	291/10114 (2.9)	Ref	644/10114 (6.4)		Ref
Middle quantile	238/10419 (2.3)	1.0 (0.9–1.3)	549/10419 (5.3)		1.1 (0.9–1.2)
Upper 33%	164/10,060 (1.6)	1.5 (1.2–1.9)	431/10060 (4.3)		1.3 (1.1–1.5)

Adjusted for: gender, age group, cohabitation, previous mental health problem or substance abuse

*N* 1266 missings for information on education are excluded

*OR* odds ratio, *CI* confidence interval (95%), *GP-MHC* general practitioner-mental health counseling (talk therapy)

Specifically, for education, aOR for GP contact was low for individuals with the highest education compared to those with the lowest educational level and even lower after the total 16 months. Having a higher education was associated with higher odds for contact to outpatient psychiatry before ID, but not after 16 months.

The highest income group had lower aOR for contact to GP before ID, compared to the lowest income group, but this difference was not significant after 16 months. High income was associated with less contact to private psychiatrist before and after index date (aOR 0.6 CI 0.5–0.7); however, the high-income group had higher aOR for contact to emergency mental health services throughout the observation period, compared to the lowest income group.

Private psychiatrist was the only type of service where income and education had directly opposite association to contact in the adjusted odds ratios.

The adjustment reversed the associations for contact to emergency mental health care and to hospital admissions; for contact to outpatient psychiatry, this was seen only prior to ID and for education.

High SEP was associated with lower rate of visits to GP, and a trend towards higher rates of visits to GP-MHC, psychologist, and private psychiatrist the four months prior to ID (Table 3). These trends had all become significant after 16 months, when, additionally, visit rates to outpatient psychiatry were higher for individuals in high SEP.

**Table 3:** Education and income. Incidence rate ratio for health care visits within 4 months index date and total 16 months

	Education					
	≤ 4 months before			16 months total		
ny	Exposure			Exposure		
Total *N*	14,182,120 days (N = 29,327 pts.)	Mean; var	aIRR (CI)	14,182,120 days (N = 29,327 pts.)	Mean; var	aIRR (CI)
GP						
< 10 years	23607/7696 (3.1)	3.1;6.7	Ref	98890/9786 (10.1)	10.1;72.2	Ref
10–12 years	28286/9681 (2.9)	2.9;5.6	1.0 (0.9–1.0)	119939/12560 (9.5)	9.5;54.0	0.9 (0.9–0.9)
12+ years	13509/4875 (2.8)	2.8;4.6	0.9 (0.9–0.9)	56141/6393 (8.8)	8.8;44.8	0.8 (0.8–0.8)
GP-MHC						
< 10 years	1499/940 (1.6)	1.6;0.9	Ref	10711/3253 (3.3)	3.3;4.3	Ref
10–12 years	2654/1541 (1.7)	1.7;1.1	1.1 (1.0–1.1)	17951/5009 (3.6)	3.6;4.7	1.1 (1.0–1.1)
12+ years	1871/1066 (1.8)	1.8;1.1	1.1 (1.0–1.1)	10570/2813 (3.8)	3.8;4.8	1.1 (1.1–1.1)
Psychologist						
< 10 years	1319/409 (3.2)	3.2;4.2	Ref	7782/1215 (6.4)	6.4;25.3	Ref
10–12 years	3277/897 (3.7)	3.7;5.9	1.1 (1.1–1.2)	20969/2697 (7.8)	7.8;32.5	1.2 (1.2–1.2)
12+ years	2513/668 (3.8)	3.8;6.4	1.2 (1.1–1.3)	16005/1858 (8.6)	8.6;33.8	1.3 (1.3–1.4)
Private psychiatrist						
< 10 years	979/391 (2.5)	2.5;4.8	Ref	6906/1003 (6.9)	6.9;35.9	Ref
10–12 years	1016/385 (2.6)	2.6;4.9	1.1 (1.0–1.2)	8100/1086 (7.5)	7.4;40.1	1.1 (1.0–1.1)
12+ years	637/241 (2.6)	2.6;4.0	1.1 (1.0–1.2)	5941/710 (8.4)	8.4;50.9	1.2 (1.2–1.3)
Outpatient psychiatry						
< 10 years	2037/582 (3.5)	3.5;12.3	Ref	19807/2037 (9.7)	9.7;136.2	Ref
10–12 years	1830/514 (3.6)	3.5;15.1	1.0 (1.0–1.1)	23508/2026 (11.6)	11.6;166.3	1.3 (1.2–1.3)
12+ years	838/214 (3.9)	3.9;19.9	1.2 (1.1–1.3)	11040/871 (12.7)	12.7;173.7	1.4 (1.4–1.5)
Emergency, short admission						
< 10 years	479/368 (1.3)	1.3;1.0	Ref	1002/635 (1.6)	1.6;2.5	Ref
10–12 years	477/395 (1.2)	1.2;0.4	1.0 (0.8–1.1)	970/702 (1.4)	1.4;0.8	0.9 (0.8–1.0)
12+ years	245/184 (1.3)	1.3;1.0	1.1 (0.9–1.2)	485/319 (1.5)	1.5;3.0	1.0 (0.9–1.1)
Hospital admission						
< 10 years	345/281 (1.2)	1.2;0.4	Ref	989/602 (1.6)	1.6;1.8	Ref
10–12 years	287/250 (1.1)	1.1;0.2	0.9 (0.8–1.1)	960/644 (1.5)	1.5;1.6	1.0 (0.9–1.0)
12+ years	143/125 (1.1)	1.1;0.2	0.9 (0.8–1.2)	453/308 (1.5)	1.5;1.1	0.9 (0.8–1.0)

	Income					
	≤ 4 months before			16 months total		
Exposure/Total *N*	14,744,394 days (*N* = 30,593 pts.)	Mean; var	aIRR (CI)	14,744,394 days (*N* = 30,593 pts.)	Mean; var	aIRR (CI)
GP						
Lower 33%	22785/7632 (3.0)	3.0;5.9	Ref	93807/9852 (9.5)	9.5;60.6	Ref
Middle quantile	24578/8009 (3.1)	3.0;6.1	1.0 (1.0–1.0)	103029/10207 (10.1)	10.0;59.9	1.0 (1.0–1.0)
Upper 33%	21079/7483 (2.8)	2.8;5.2	0.9 (0.9–0.9)	89973/9808 (9.2)	9.1;54.2	0.9 (0.9–0.9)
GP-MHC						
Lower 33%	1777/1103 (1.6)	1.6;0.9	Ref	11435/3499 (3.3)	3.3;4.5	Ref
Middle quantile	2099/1210 (1.7)	1.7;1.1	1.0 (1.0–1.1)	13961/3877 (3.6)	3.6;4.7	1.1 (1.1–1.1)
Upper 33%	2332/1338 (1.7)	1.7;1.1	1.0 (1.0–1.1)	14994/4059 (3.7)	3.7;4.6	1.1 (1.1–1.1)
Psychologist						
Lower 33%	1813/506 (3.6)	3.6;5.7	Ref	10564/1432 (7.4)	7.4;33.4	Ref
Middle quantile	2547/698 (3.6)	3.6;6.2	1.1 (1.0–1.1)	15434/2010 (7.7)	7.7;32.4	1.1 (1.0–1.1)
Upper 33%	2862/801 (3.6)	3.6;5.6	1.1 (1.0–1.2)	19429/2429 (8.0)	8.0;30.6	1.1 (1.1–1.2)
Private psychiatrist						
Lower 33%	1289/532 (2.4)	2.4;4.4	Ref	9303/1307 (7.1)	7.1;38.9	Ref
Middle quantile	848/307 (2.8)	2.8;4.8	1.2 (1.1–1.3)	6493/849 (7.6)	7.6;43.4	1.1 (1.0–1.1)
Upper 33%	610/227 (2.7)	2.7;4.3	1.2 (1.1–1.3)	5901/754 (7.8)	7.8;41.1	1.1 (1.1–1.2)
Outpatient psychiatry						
Lower 33%	2605/687 (3.8)	3.8;15.2	Ref	25132/2256 (11.1)	11.1;163.6	Ref
Middle quantile	1579/453 (3.5)	3.5;14.5	1.0 (0.9–1.0)	18172/1702 (10.7)	10.7;139.5	1.0 (1.0–1.1)
Upper 33%	748/234 (3.2)	3.2;11.6	0.9 (0.8–1.0)	13124/1169 (11.2)	11.2;166.6	1.1 (1.1–1.2)
Emergency, short admission						
Lower 33%	527/412 (1.3)	1.3;0.6	Ref	1050/696 (1.5)	1.5;1.9	Ref
Middle quantile	418/321 (1.3)	1.3;0.9	1.0 (0.9–1.2)	872/561 (1.6)	1.5;2.4	1.0 (0.9–1.1)
Upper 33%	314/256 (1.2)	1.2;0.7	1.0 (0.9–1.2)	630/463 (1.4)	1.4;1.1	1.0 (0.8–1.1)
Hospital admission						
Lower 33%	343/289 (1.2)	1.2;0.3	Ref	1001/642 (1.6)	1.6;1.6	Ref
Middle quantile	289/236 (1.2)	1.2;0.4	1.0 (0.9–1.2)	906/546 (1.7)	1.7;2.1	1.1 (1.0–1.2)
Upper 33%	183/163 (1.1)	1.1;0.2	1.0 (0.8–1.2)	586/426 (1.4)	1.4;0.7	1.0 (0.9–1.1)

Adjusted for: gender, age group, cohabitation, previous mental health problem or substance abuse

*IRR* Incidence rate ratio, *CI 95%* confidence interval, *aIRR* adjusted IRR, *var.* variance, *GP-MHC* general practitioner-mental health counseling (talk therapy)

**Fig. 1:**
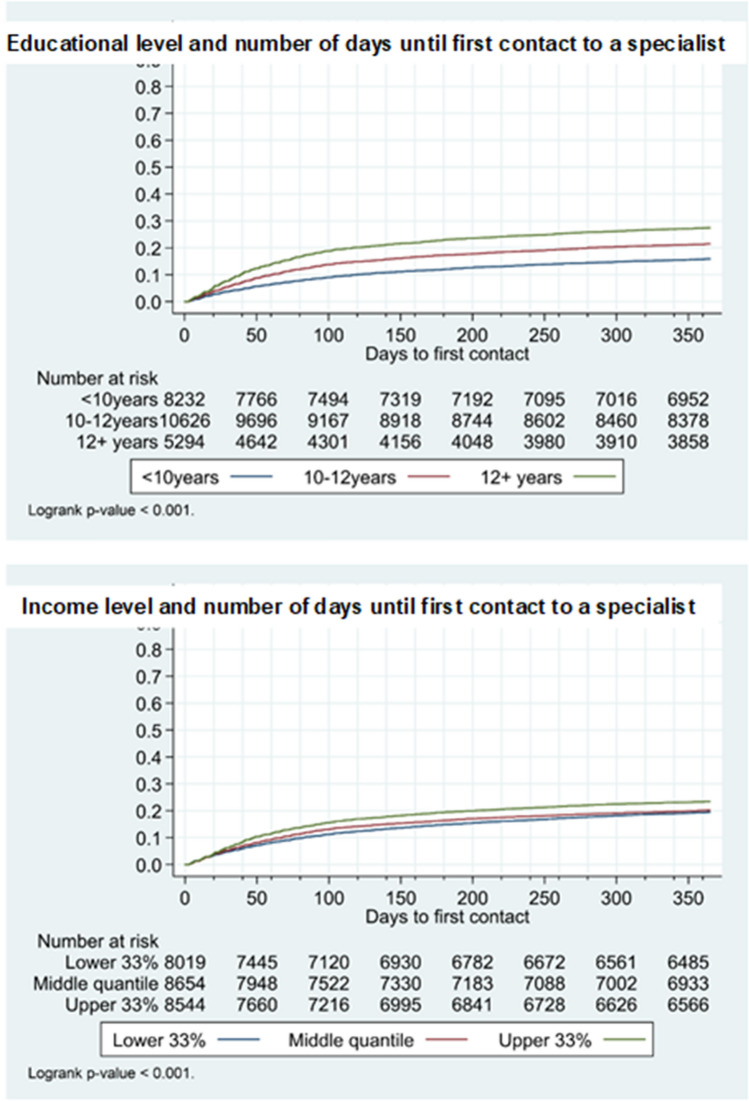
Kaplan–Meier graphs

25,217 individuals used no specialized services before ID and were analyzed with respect to time to contact to psychologist or psychiatrist. The Kaplan–Meier graphs (Fig. 1) for the time to event in in the 365 days after ID show 10% had contact to psychologist or psychiatrist within 40 days after ID if they had a higher education; whereas, 10% of those with a short education would reach specialized care by day 120. At that time (day 120), 20% of the individuals with the highest educational level was in contact with that type of care. As for income, 10% in the highest income group had contact to specialized services within approximately 45 days; whereas, 80 days had passed before the same proportion in the lowest income group had established contact.

The estimated hazard ratios for contact to psychologist or psychiatrist for the same 25,217 individuals in the 12 months after ID (Table 4) showed that the group with the highest educational level gained contact to specialized services at almost twice the rate as the shortest (adjusted hazard ratio (aHR) 1.9 CI 1.8–2.1). Income also had an impact on contact rates, as the highest income group were 40% more likely to have contact than the lowest income group, in the adjusted analyses.

**Table 4:** Hazard ratios for contact to psychologist or psychiatrist after ID, by level of education and income

	HR crude	HR adjusted
Education		
< 10 years	Ref	Ref
10–12 years	1.4 (1.3–1.5)	1.4 (1.3–1.5)
12 + years	1.9 (1.7–2.0)	1.9 (1.8–2.1)
Income		
Lower 33%	Ref	Ref
Middle quantile	1.1 (1.0–1.1)	1.1 (1.1–1.2)
Upper 33%	1.2 (1.2–1.3)	1.4 (1.3–1.5)

## Discussion

This nationwide sample of initial users of antidepressants included considerable higher proportions of individuals with short education and temporarily out of job than the population it was sampled from—and as such showed a heavy social bias by the inclusion criteria alone. Except for consultations at GP, we have found a social gradient favoring patients in high SEP in all areas in the adjusted analyses, before and after ID, most explicit when measured by education. However, contact to private psychiatrist was an exception to this for the lowest income group.

To be in high SEP was associated with higher chance of contact—and two to three times more rapidly contact to a psychologist or psychiatrist after initiated treatment with antidepressant and no previous mental health care treatment; most evident when measured by education. To our knowledge, this type of study, analyzing MHCU before and after initiated AD treatment based on the high-quality register data, has not been reported before, nor have we been able to locate studies on socioeconomic disparity in time to access specialized care as psychologist or psychiatrist.

### Strengths and limitations

A strength of this study is the nationwide sampling which enable us to capture a greater part of individuals in low SEP, a problem in public surveys. Another advantage is the use of high-quality public registers [24] and gain of detailed and reliable information on health care contacts and avoidance of the problem of recall bias [25]. However, some patients may not be included if they have payed to full expense for psychologist or psychiatrist. We do not know the extent of such practice, but expect it to be low; nevertheless, this factor only adds to the direction of socioeconomic disparity. Another advantage is the combination of health registers with the CRS, giving accurate data on individual status and almost none lost to follow-up.

As for selection bias, initial use of antidepressants does not classify subjects as being depressed or having a common mental disorder, per se. We expect our study will include some off-label prescriptions. A study on middle-aged and elderly population reports 15% of the SSRI-treated individuals are of unknown or off-label indications [26]. A large Swedish study reports 81% diagnosed with major depression are treated in primary care only (by GP or psychologist), whereof 76% receive antidepressants [27]. This could indicate a low threshold for treatment with antidepressants, and thus may have an impact on who are included in our sample, but the association to SEP is not known. It is also possible that some individuals included are not depressed; however, we would expect very few non-depressed individuals to be referred to specialized services.

Not all depressed persons are treated with antidepressants and therefore, not all depressed persons are included in our study; some may not use any health care services at all, and some may have been treated by psychologist or by GP-MHC without any further need for antidepressants. In a previous study, we found that 10% of individuals with moderate to severe symptoms of depression did not contact their GP 6 months following the scoring, and another 47% in contact with their GP had no treatment; neither of these were found to have association with SEP [28]. The detection rate of depressive disorders without psychometric tests is low by GP [29].

As for information bias, we do not know the severity of the disorder; we assume the prescription pattern is the same across the socioeconomic groups.

Some findings were unexpected and notable, first, the sample itself—in gender constellation; second, the association between SEP and hospitalization for mental health care. We will address these initially and our study objectives afterwards.

### Gender

The female–male ratio was notable; commonly, the ratio is reported to be almost 2:1 for depressive and anxiety disorders [30, 31]; with 57% females, we found a modest gender difference. A male–female proportion at 3:5 was reported in a recent study as the incidence rate of pharmacologically treated depression among individuals 15–44 years of age in Denmark [32]. This could be a sign of selection bias if men are more often treated with antidepressants compared to women; yet, a Swedish survey study on gender differences in self-reported depression and prescribed antidepressants finds men to report depression to a greater extent than women but prescribed antidepressants to a lesser extent; whereas, women are prescribed antidepressants without reporting depression [33]. Similarly, a Danish study reports men to have lower use of antidepressants compared to women, when in poor mental health [34]. Culture may be a part of the explanation, as a study from Belgium on gender difference in treatment preferences suggests. They find men to report less positive attitudes toward the helpfulness of psychotherapy, and women to consider psychotherapy as less helpful for men, and recommend self-care to men with mental health problems [35]. In the present study, the female–male contact ratio to psychologists was 3:2, also prior to ID, whereas there was no difference in contact ratio to private/public psychiatrists; however, hospital admissions were dominated by males.

### Hospitalization

We found high SEP associated with higher odds (aOR 1.3 CI 1.1–1.5) for admission to hospital before and after ID. This association is unexpected, since individuals with depression and low economic resources are reported to have a higher risk for admission to psychiatric hospital care [36]. Suicide risk is a common reason for hospital admission for depressed patients and may explain a part of this. A Finnish study on longitudinally risk factors for suicide after first hospitalization with depression find higher family income, and higher education to predict future suicide, plus male sex, previous suicide attempts and severity of depression [37]. Likewise, a Swedish register-based study on health care use of workers in sickness absence due to common mental disorders finds that risk estimates for high education and subsequent inpatient care due to mental disorders or suicide attempts are higher compared to estimates for patients with lower education [38]. We found psychiatric comorbidity and male sex associated with higher odds for hospital admission. A possible explanation for this could be that males have more severe symptoms due to delays in receiving care [39].

### Socioeconomic difference in the health care use before and after initiating antidepressant treatment

Almost one in four had no GP contact before ID or any other registered contact to the services followed in our sample (sup Table 2). This may be due to prescription by other types of medical doctors (somatic hospital or outpatient clinics). Others report 15% are first treated with antidepressants in a hospital setting in the age group 15–44 [32].

Danish and international treatment guidelines [40] recommend AD treatment to be preceded by a stepped care, with psychoeducation and psychological interventions as the first steps before pharmacological treatment; this was not reflected in our data. When including all individuals in contact with psychologist prior to ID (6.6%) and those who received GP-MHC (12%) in the same period, evidently few had psychoeducation or psychological interventions before initiating their antidepressant treatment (sup Table 1).

A weak adaption of the stepped care approach is observed in another Danish study [41], combining national survey data of more than 100,000 participants with scores on the Perceived Stress Scale with national registers on health care use. Among respondents in the highest stress quintile, 6.8% attend GP-MHC, 3.3% consult a psychologist, and 21.5% redeem an antidepressant prescription. The authors reason that persons with stress and physical multimorbidity might not have the mental resources needed to interact in psychological treatment and may choose pharmacological treatment or may be recommended so by their GP.

Though not considering multimorbidity, we found high SEP to be associated specifically with more GP-MHC and psychologist services both before and after initiating antidepressant treatment.

After ID, almost 28% did not reimburse an antidepressant a second time the following year. This group was dominated by individuals with previous public mental health care treatment, more males, more in low SEP and more unemployed. Some may have switched to TCA, but this was not possible to reveal with data at hand. A high-proportion discontinuing initial antidepressant treatment is not unusual. Burton et al. report 25% initiating antidepressant treatment by GP has a treatment period of 30 days or less, the group dominated by individuals from deprived areas and younger [42].

Before ID, higher SEP was associated with more contact to GP-MHC, psychologist, emergency care and hospital admissions; after additional 12 months, the discrepancy was uphold but faded—except for contact to psychologist where the disparity widened over time. Others report similarly, that across Europe, higher SEP groups are more likely to use health care specialists, compared with groups in lower SEP, with a tendency that countries with higher or equal probability of GP utilization by lower SEP groups have more consistent probability of specialist use in high-SEP groups [43]. We found the same tendency for MHCU.

For the rate of visits—among those who had contact to the specific type service—we saw the same pattern, though the socioeconomic difference turned from not significant before ID to significant in favor of higher education or income after the full observation period. Notably, the rates of visits to private psychiatrist were higher for high-income groups before as well as after ID. If low-income individuals use a free private psychiatrist instead of a co-pay psychologist, as seen in higher OR for contact, their frequency of visits may well be lower, given their needs thereby could be lower as well. Additionally, distance has a stronger negative impact on MHCU for patients in low SEP [12].

### Socioeconomic difference in time to first contact to psychologist or psychiatrist

More than 80% had no contact to specialized services prior to ID; following them 12 months afterwards, we found a clear difference between socioeconomic groups in time to first contact to psychologist or psychiatrist, favoring patients with a higher education in the adjusted analyses. It took twice as long for the lowest compared to the highest educated individuals to get contact with a specialist, most pronounced within the first 100 days.

When high SEP is associated with not only higher odds for contact to specialized services but also much faster access, it could be linked to abilities associated with being in high SEP or referral practice by GP.

Waiting times for somatic health services have an unequal impact on socioeconomic groups as significantly longer waits are found for patients in low compared to patients in high SEP [44]. This is likely to apply to mental health care as well and may even be aggravated, due to the initiative needed to get access which may be more difficult to mobilize for patients with mental health problems. Low SEP was associated with higher odds for contact to private psychiatrists compared to psychologists and difference in waiting time to these services could explain the difference. However, there is no substantial difference in waiting time for these services [45, 46].

The GP will refer to specialist services, but the patients must arrange the appointment by them self—except when referred to outpatient services (public) where the patient is called in after a referral. Short education is found to be associated with doubt about how to get professional care among individuals with symptoms of depression [47]; which can explain some of the lower odds for individuals with a short education—versus the low-income group—to use private psychiatrists as well psychologist.

Ability to pay and co-payment could explain the pattern of psychologist use, shown by double odds for contact by high-income group compared to the lowest income group and almost triple odds for highest education compared to lowest on contact to psychologist. Ability to seek care and social norms could also explain the association with education, as stigmatizing attitudes can influence health care usage. However, we did not find an association between stigma and SEP in our previous study or in the literature [47].

Beyond GP consultation, we found a social gradient in MHCU favoring patients in high SEP in all areas in the adjusted analyses, except for contact to private psychiatrist where the lowest income group had much higher odds for contact. We expect co-payment to psychologist to explain this difference, given the diagnostic overlap of patients in these two types of mental health care and the known socioeconomic effect of co-payment [48]. It is inequity in mental health care per se, when evidence-based care is tailored by co-payment when delivered by psychologists. This paper shed light on this and the diversion of care it seems to cause.

We found treatment contact was predominantly associated with education rather than income, in line with a recent systematic review on health services use for common mental disorders [49].

## Conclusions

Being in high SEP was associated with less use of GP and increased the use of all MHC services in the period before initiated treatment with antidepressants and afterwards as well.

Co-payment for psychologist services seems to be compensated by a diversion of the MHCU towards free private psychiatrist for the low-income group.

Access to psychologist and psychiatrist happened much faster for individuals in high SEP than for individuals in low SEP, when adjusted for sex, age, cohabitation status and previous mental health or addiction disorder.

### Clinical implications

The most consistent socioeconomic discrepancy was seen in contact to GP-MHC and psychologist. Whereas the psychologist can be explained by co-payment, the provision of GP-MHC may indicate suboptimal care.

The stepped care approach on treatment of depression does not seem to be practiced. Reconsidering the relevance of the stepped care or initiatives to encourage it ought to be considered.

The GP may consider a more proactive approach in the care of patients with depressive/anxiety disorders and short education—by earlier referral to specialist services. These patients may also need concrete advice on how and where to access care.

## Electronic supplementary material

Below is the link to the electronic supplementary material.

Supplementary file1 (XLS 48 kb)

Supplementary file2 (DOCX 14 kb)

Supplementary file3 (XLS 34 kb)

Supplementary file4 (XLS 33 kb)

Supplementary file5 (XLS 47 kb)
