# Supplementary material for: Socioeconomic position and mental health care use before and after first redeemed antidepressant and time until subsequent contact to psychologist or psychiatrists: a nationwide Danish follow-up study
Source: Soc Psychiatry Psychiatr Epidemiol. 2020 Jul 8;56(3):449–62. doi: 10.1007/s00127-020-01908-7 (PMC7904708; doi:10.1007/s00127-020-01908-7)
Supplement: Supplementary file 2 — Supplementary file2 (DOCX 14 kb) [file 127_2020_1908_MOESM2_ESM.docx]

***Supplementary 1. Supplement to method***

***Independent variables***

In this study, we used equivalent disposable family income, which adjusts the family income according to number of family members and children under the age of 25 years living at home after payment of taxes and interest. (The first adult counts as 1, each consecutive person aged >14 years as 0.5, and each person aged 0-14 years as 0.3. For a family of two adults and two children aged < 14 years, the household income will be divided by 2.1). The income was categorized into five groups according to quintiles.

***Dependent variables***

*Information related to treatment*

GP-MHS is talk therapy provided by a GP. There is no formal requirement to the methods, except that it should be relevant .The GP has to receive regular supervision – from other GPs, psychologists or psychiatrists individually or in groups(2). Data were drawn from The Danish National Health Service Register for Primary Care, according to the codes in table S1 (3).

*Table S1: Codes for services provided in primary care.*

| **Type of health care service** | **Code in The Danish National Register for Primary Care** |
| --- | --- |
| GP-MHS (talk therapies) | 804003 +(804021-804027) + (804247 – 804249) + 806101 |
| Psychologist contacts | (630110 – 630211) + (630214 – 630340) |
| Psychiatrist consultations | (240110 – 240140) + (240210 – 240236) + 241401 |

The public part of the expense for a psychologist (or a psychiatrist) is covered by the public health service system, which is also the case for insured persons. Thus, privately insured persons were also included in our data. In 2013, 1.8 mill people (50% of Danes aged between 20 – 70 years) were covered by an additional private health insurance, and 13 million DKK (1.74 million €) were payed to cover expenses for psychiatrists or psychologists(4).

Reference list

(1) Vestergaard P, Rejnmark L, Mosekilde L. Osteoporosis is markedly underdiagnosed: a nationwide study from Denmark. Osteoporos Int 2005 February;16(2):134-41.

(2) RLTN. Forhandlingsaftale 2010 RLTN og PLO. 1-83. 21-12-2010. Ref Type: Generic

(3) Andersen JS, Olivarius NF, Krasnik A. The Danish National Health Service Register. Scand J Public Health 2011 July;39(7 Suppl):34-7.

(4) forsikringogpension. Flere-end-to-millioner-danskere-har-nu-en-sundhedsforsikring.aspx. [www.forsikringogpension.dk](http://www.forsikringogpension.dk) . 11-1-2016. Ref Type: Online Source
